# Supplementary material for: Deletion of wfs1 Impairs Oligodendrocyte Precursor Cells Dorsal Distribution and Myelination Through the wfs1-hmgcs1 Axis in Zebrafish
Source: Biology (Basel). 2026 Aug 16;15(16):1402. doi: 10.3390/biology15161402 (PMC13510151; doi:10.3390/biology15161402)
Supplement: Supplementary file 1 [file biology-15-01402-s001.zip › File S1-Transcriptome sequencing data and differentially expressed genes (DEGs) in wfs1-- zebrafish at 5 dpf/1.GO/wfs1KOvsWT/GSEA/index.html]

Index for xtools.gsea.Gsea wfs1KOvsWT.Gsea.1700447271245


### GSEA Report for Dataset deg

#### Enrichment in phenotype: **wfs1KO (4 samples)**

- 2000 / 4072 gene sets are upregulated in phenotype
   **wfs1KO**
- 131 gene sets are significant at FDR < 25%
- 252 gene sets are significantly enriched at nominal pvalue < 1%
- 376 gene sets are significantly enriched at nominal pvalue < 5%
- Snapshot
  of enrichment results
- Detailed
  enrichment results in html
  format
- Detailed
  enrichment results in excel
  format (tab delimited text)
- Guide to
  interpret results

#### Enrichment in phenotype: **WT (4 samples)**

- 2072 / 4072 gene sets are upregulated in phenotype
   **WT**
- 18 gene sets are significantly enriched at FDR < 25%
- 202 gene sets are significantly enriched at nominal pvalue < 1%
- 323 gene sets are significantly enriched at nominal pvalue < 5%
- Snapshot
  of enrichment results
- Detailed
  enrichment results in html
  format
- Detailed
  enrichment results in excel
  format (tab delimited text)
- Guide to
  interpret results

#### Dataset details

- The dataset has 31891 features (genes)
- No probe set => gene symbol collapsing was requested, so all 31891 features were used

#### Gene set details

- Gene set size filters (min=15, max=5000) resulted in filtering out 10409 / 14481 gene sets
- The remaining 4072 gene sets were used in the analysis
- List of
  gene sets used and their sizes
  (restricted to features in the specified dataset)

#### Gene markers for the **wfs1KO** *versus* **WT** comparison

- The dataset has 31891 features (genes)
- # of markers for phenotype
   **wfs1KO** 
  : 15957 (50.0% ) with correlation area 53.2%
- # of markers for phenotype
   **WT** 
  : 15934 (50.0% ) with correlation area 46.8%
- Detailed
  rank ordered gene list
  for all features in the dataset
- Heat map and gene list correlation
  profile for all features in the dataset
- Buttefly plot
  of significant genes

#### Global statistics and plots

- Plot of
  p-values
   *vs.* 
  NES
- Global ES
  histogram

#### Other

- Parameters
  used for this analysis

#### Comments

- Timestamp used as random seed: 1700447419530
- Warning: Phenotype permutation was performed but the number of samples in class A is < 7, phenotype: sample.cls
- Warning: Phenotype permutation was performed but the number of samples in class B is < 7, phenotype: sample.cls
- With small datasets, there might not be enough random permutations of sample labels to generate a sufficient null distribution. In such cases, gene\_set randomization might be a better choice.

---

Report: wfs1KOvsWT.Gsea.1700447271245.rpt   by user: rnaai

xtools.gsea.Gsea [Mon, Nov 20, '23 10 AM 27]

Website:
www.gsea-msigdb.org/gsea
Questions & Suggestions:
Contact page
